# Long Noncoding RNAs in Digestive System Malignancies: A Novel Class of Cancer Biomarkers and Therapeutic Targets?

**DOI:** 10.1155/2015/319861

**Published:** 2015-04-29

**Authors:** Athina Kladi-Skandali, Kleita Michaelidou, Andreas Scorilas, Konstantinos Mavridis

**Affiliations:** Department of Biochemistry and Molecular Biology, University of Athens, 15701 Athens, Greece

## Abstract

High throughput methodologies have revealed the existence of an unexpectedly large number of long noncoding RNAs (lncRNAs). The unconventional role of lncRNAs in gene expression regulation and their broad implication in oncogenic and tumor suppressive pathways have introduced lncRNAs as novel biological tumor markers. The most prominent example of lncRNAs application in routine clinical practice is PCA3, a FDA-approved biomarker for prostate cancer. Regarding digestive system malignancies, the oncogenic HOTAIR is one of the most widely studied lncRNAs in the preclinical level and has already been identified as a potent prognostic marker for major malignancies of the gastrointestinal tract. Here, we provide an overview of recent findings regarding the emerging role of lncRNAs not only as key regulators of cancer initiation and progression in colon, stomach, pancreatic, liver, and esophageal cancers, but also as reliable tumor markers and therapeutic tools. lncRNAs can be easily, rapidly, and cost-effectively determined in tissues, serum, and gastric juice, making them highly versatile analytes. Taking also into consideration the largely unmet clinical need for early diagnosis and more accurate prognostic/predictive markers for gastrointestinal cancer patients, we comment upon the perspectives of lncRNAs as efficient molecular tools that could aid in the clinical management.

## 1. Introduction

Gastrointestinal (GI) cancer is an umbrella term which refers to a diverse group of tumors that affect the digestive system and accessory organs, including the esophagus, stomach, gallbladder, liver, pancreas, and small and large intestine [1]. According to recent GLOBOCAN data, 4.06 million new cases and 3.03 million deaths of GI cancer occurred in 2012, worldwide. Liver (LC), gastric (GC), and colorectal (CRC) cancers are ranked second to fourth in terms of worldwide cancer-related mortality, behind only lung cancer [2]. Pancreatic cancer incidence is increasing rapidly and prognosis remains poor, with a 5-year survival rate of as little as 6% [3]. Esophageal cancer is a virulent malignancy of the upper GI tract and overall 5-year relative survival remains below 20% [4].

One of the common features of digestive system carcinomas, which can partially explain the abovementioned dismal statistics, is the lack of early alarming clinical symptoms. Early neoplastic lesions are macroscopically similar to normal tissues and this complicates their detection with endoscopic and/or imaging approaches [1]. Adding to this perplexity, robust diagnostic biomarkers for most GI cancers are not yet available. For instance, even serum carbohydrate antigen 19-9 (CA19-9) has relatively poor specificity and sensitivity, to warrant its use as a screening biomarker for pancreatic cancer [1, 5]. Furthermore, a large proportion of GI cancer patients who have undergone treatment, suffer from frequent relapse and metastatic recurrence of disease. Unpredictable chemotherapy resistance is another major contributor to the poor clinical outcome of advanced stage GI cancer patients [6–8].

An in-depth understanding of the previously uncharted molecular pathways that promote the multistep process of GI cancer initiation, progression, and chemoresistance could be effectively translated into biomarkers that will accelerate the realization of an optimal clinical management for GI cancer patients. One such molecular mechanism, as suggested by continuously growing body of evidence, is the regulation by long noncoding RNAs (lncRNAs), a species of RNA molecules that are steadily becoming the next frontier for cancer translational research.

## 2. Long Noncoding RNAs: An Unconventionally Unique Component of the Human Transcriptome

The application of high-throughput sequencing technologies and bioinformatic methods, uncovered the existence of an extraordinary number of non-protein coding RNAs (ncRNAs) expressed by the human genome [9]. These untranslated RNA transcripts are categorized into short and long ncRNAs according to their size, which in turn, can be divided into subclasses based on their functional and structural features [10]. Although research interest has long been monopolized by short ncRNAs, such as microRNAs, a lot of attention is now focused towards the abundant and diverse class of lncRNAs.

lncRNAs are endogenous cellular RNA molecules more than 200 nucleotides long, which do not possess open reading frames (ORFs) of significant length. Although compelling evidence shows that lncRNAs lack protein-coding capacity, it is possible that a subset of lncRNAs with small ORFs, may indeed code for short peptides [11, 12]. lncRNAs are predominantly transcribed by RNA polymerase II and are subsequently subjected to 5′-end capping, 3′-end polyadenylation, and splicing. However, multiple lncRNAs are produced by RNA polymerase III and remain nonpolyadenylated [13]. lncRNAs are localized to the nucleus and cytoplasm where they can interact with DNA, RNA, and proteins and act as gene expression regulators at the transcriptional and/or posttranscriptional levels. Moreover, lncRNAs display tissue-, cell-, developmental-, and disease-specific expression patterns [14] and are detectable in several bodily fluids, such as blood and urine [15].

Recently, Ma et al. [16] thoroughly devised lncRNAs according to their genomic location, their distance of the target gene, the targeting mechanisms, and their exact mode of action. Based on their genomic position, lncRNAs are characterized as sense, antisense, intronic, and intergenic lncRNAs (lincRNAs). A lncRNA molecule can be either a* cis*-acting agent, regulating the expression of a neighbor gene of the same chromosome, or a* trans*-acting element, inducing epigenetic changes on a distant gene of the same or different chromosome. In terms of targeting mechanisms, lncRNAs act as (1) markers for the spatiotemporal expression of the target genes (signal), (2) adaptors for the assembling of functional protein complexes (scaffold), (3) factors that tether specific proteins and determine the proper localization of the resultant complex (guide), and (4) molecules that prevent the interaction of another RNA or protein with its natural targets (decoy). Based on their mechanism of action, lncRNAs are categorized into three subgroups that regulate gene transcription, posttranscriptional events, and other procedures such as RNA interference [16].

## 3. Noncoding Does Not Mean Nonfunctional: Mechanisms of Action of lncRNAs and Their Role in Physiology

The first human lncRNA gene, H19, was cloned and sequenced by Brannan et al., in 1990 [17]. A year later, the X-inactive-specific transcript (XIST) was identified [18]. Currently, the estimated number of lncRNAs genes in the human genome is 56 018 [19], the exact functions of which are only beginning to be unraveled. lncRNAs are considered as ideal orchestrators of RNA-based regulatory mechanisms, adding a new layer to the, already complex, network of gene expression control. Although information regarding the exact mechanisms of action of lncRNA remain scant, mounting experimental data suggest that they are involved in each step of the gene information processing,* via* distinct mechanisms [20].

lncRNAs play crucial roles in epigenetic regulation of gene expression. In this case,* cis*- or* trans*-acting lncRNAs (e.g., XIST and HOTAIR, resp.), serving as scaffold molecules, direct the recruitment of histone or chromatin modifying complexes to the target gene and induce its repression. Besides, activating lncRNAs, known as enhancer-associated lncRNAs, mediate the remodeling of chromatin landscape so as active enhancers to be gathered in close proximity to the target gene (e.g., HOTTIP) [21, 22].

Distinct mechanisms have been described regarding the function of lncRNAs in transcriptional regulatory programs. For example, these molecules are able to interact with a variety of transcription factors and to promote their activation/inactivation or to determine their subcellular localization. Moreover, lncRNAs can inhibit Pol II or conversely facilitate the binding of this enzyme to the gene promoter [23]. lncRNAs can also regulate gene expression in the posttranscriptional level. More precisely, lncRNAs act as miRNA “sponges” where, the lncRNA-miRNA base pairing modifies the latter's posttranscriptional effect and alters the expressional profile of their mRNA-target (e.g., HULC) [24]. Additionally, lncRNAs are implicated in alternative mRNA splicing. The most prominent example is the lncRNA MALAT1, which is proposed to interact with and control the activity of serine/arginine-rich protein, a central component of the splicing machinery [20].

lncRNAs have emerged as fine-tuners of cell differentiation and are implicated in various events in the life cycle of eukaryotes, ranging from gametogenesis to the formation of multicellular tissues and regulation of homeostasis. lncRNAs are also major parts of the transcriptional networks that induce or maintain ESC (Embryonic Stem Cells) pluripotency [25, 26]. Moreover, lncRNAs are involved in embryogenesis, since they are essential for dosage compensation (e.g., XIST), genomic imprinting (e.g., Kcnq1ot1), and the control of Hox gene expression [26]. The multifunctionality of lncRNAs allows them to take part in the complicated regulatory programs that govern the differentiation and development of distinct organs and tissues such as the central neural system, muscles, mammary gland, heart, skin, and adipose tissue [21, 25, 26].

## 4. The Extensive Implication of lncRNAs in Cancer Pathobiology: Can It Be Translated to Clinical Practice?

The established involvement of lncRNAs in a wide range of biological procedures has ushered in a new era of molecular genetics, where previously uncharacterized molecules seem to contribute to the etiology of human diseases. Modifications in the structure of lncRNAs, which are induced by large- or small-scale mutations, as well as their abnormal expression, have been linked with the onset of several pathological conditions and, most importantly, cancer [27, 28]. The aberrant expression of lncRNAs, across both solid and human hematological malignancies, brought these molecules to the forefront of cancer research and accelerated the investigation of their mechanistic roles in these multifactorial disorders. Dysregulated lncRNAs promote neoplastic transformation through the malfunction of common biological procedures such as epigenetic and transcriptional regulation, apoptosis, and epithelial-mesenchymal-transition (EMT). Although many functional aspects of lncRNAs still need to be clarified, accumulating* in vitro* and* in vivo* studies strengthen their role as oncogenes (e.g., HOTAIR, ANRIL, MALAT1, SRA, HULC, UCA1, PCA3, PCAT-1, PCGEM1, and PRNCR1) or tumor suppressors (LincRNA-p21, GAS5, MEG3, TERRA, PANDA, and TUG1) [29].

Several lncRNAs with oncogenic potential have already been reported. For instance, HOTAIR, promotes metastasis* via* a polycomb repression complex 2- (PRC2-) dependent pathway, simultaneously mediating the repression of several metastasis-suppressor genes and the activation of metastasis-enhancing genes [30]. A recent study indicates that HOTAIR may serve as regulator of EMT [31] and,* vice versa*, its expression is controlled by signaling pathways and molecules that control EMT (WNT/*β*-catenin signaling pathway and TGF-b) [29]. Similar to HOTAIR, ANRIL guides a PRC-mediated silencing of the INK4b/ARF/INK4a locus. These genes encode for three tumor suppressor proteins (p15, p14, and p16) which play crucial roles in cell cycle block, senescence, and stress-induced apoptosis. Abnormal silencing of the aforementioned genes by ANRIL is a possible mechanism for cancer initiation. MALAT1 is also implicated in metastasis; chromosomal translocation is the main mechanism behind its marked upregulation in cancer [15]. Malfunction of MALAT1 affects cancer cells' mobility, invasiveness, and survival through the abnormal splicing of genes that are involved in oncogenesis- and metastasis-related procedures (e.g., WNT and MAPK signaling, cytoskeletal organization and cell cycle, EMT) [29, 32].

Contrariwise, tumor-suppressor lncRNAs have also been described. The currently known tumor suppressor lncRNAs are mainly implicated in apoptosis and cell cycle control, albeit they are characterized by dissimilar modes of action. For example, Linc-p21, PANDA, and TUG1 are involved in the p53 signaling and thus control the expression of genes that are related with apoptosis and cell cycle [33]. MEG3 also facilitates p53 signaling by activating and promoting p53 binding to the target genes. A p53-independent mode of action, which includes the control of* Rb* and the inhibition of cell proliferation, was also described recently for MEG3 [29]. GAS5 influences cell survival fate by activating the apoptotic machinery. Specifically, GAS5 competes with Glucocorticoid Response Elements (GRE) for binding to the glucocorticoid receptor (GR) and induces transcriptional suppression of GR-related genes that act as inhibitors of apoptosis [34].

Apart from their regulatory role in oncogenic and tumor-suppressor pathways, the tissue- and cancer-specific expression patterns that lncRNAs commonly demonstrate [15] along with the advances in high-throughput expression analysis technologies have paved the way for the exploitation of these molecules in the clinical setting of oncology. Indeed, a bulk of experimental data denotes not only the dysregulation of lncRNAs in various cancers but also their association with patients' prognosis and response to treatment, rendering lncRNAs as an untapped source of diagnostic, prognostic, and predictive markers as well as a novel class of therapeutic targets.

The greatest example of the dynamics of lncRNAs as diagnostic biomarkers is the 2012-FDA approved PCA3 urine test for prostate cancer [35, 36]. Interestingly, the lncRNA UCA1 (Urothelial Carcinoma Associated 1) can also be detected in urine sediment and represents a promising noninvasive diagnostic marker of bladder cancer [37]. Investigating the potential role of lncRNAs as prognostic and predictive markers has already provided encouraging results. In particular, HOTAIR is upregulated in both primary and metastatic breast tumors and is a robust marker of unfavorable prognosis regarding overall survival (OS) and metastasis [32]. MALAT1 is significantly overexpressed in metastasizing non-small cell lung cancer (NSCLC) tumors and serves as an independent prognostic indicator of poor survival for stage I NSCLC patients [38]. Moreover, H19 is a potent predictor of early recurrence in bladder cancer patients [24]. Expectations have also been raised regarding the potential of lncRNAs as predictive markers. For instance, XIST is correlated with the therapeutic response in ovarian cancer [15], and MALAT1 expression in osteosarcoma is correlated with poor response to chemotherapy [39].

The possible application of lncRNA-based therapies in clinical practice has attracted much pharmaceutical research interest and the first clinical trials are already underway (e.g., the DTA-H19 vector in bladder, ovarian, and pancreatic cancer) [40, 41]. Another approach, regarding the use of lncRNAs in cancer therapy, is the combined use of siRNAs against certain lncRNAs with conventional chemotherapeutic agents, which seems to result in effective sensitization of tumors to the latter [33, 42]. Antisense oligonucleotides represent an alternative option to achieve lncRNA targeting and recent studies demonstrated that this strategy can result in inhibition of MALAT1 and arrest of metastasis in mouse models [42].

## 5. lncRNAs as a Novel Class of Cancer-Related Molecules and Novel Biomarkers in Digestive System Malignancies

An exponentially increasing number of studies report that lncRNAs are deregulated in GI neoplasms. Analyses of aberrantly expressed lncRNAs, though mechanistic studies, provide new insights into their ubiquitous implication in pathways that govern hallmark processes of cancer, including cell proliferation, evasion of apoptosis, tumor invasion, and metastasis.

### 5.1. Mechanistic Aspects of lncRNA Involvement in Digestive System Malignancies

**Figure 1:**
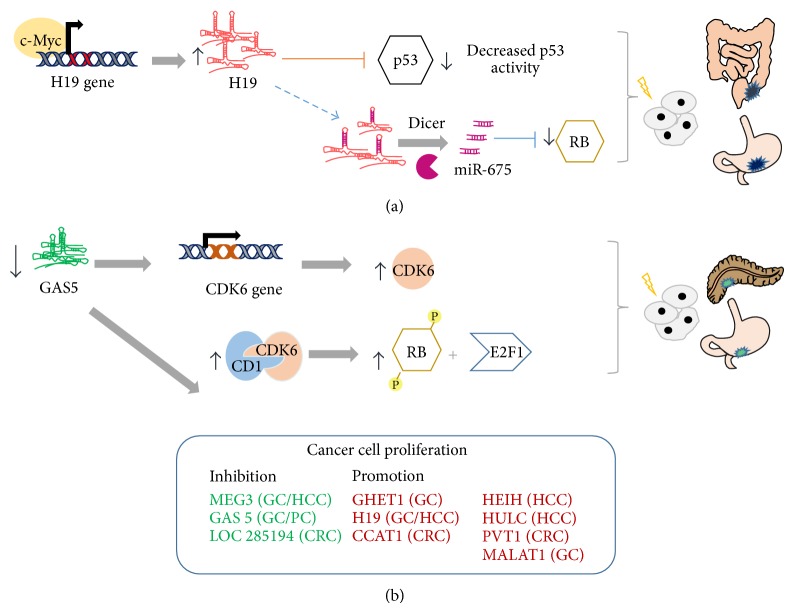
Representative mechanisms of lncRNAs implication in gastrointestinal cancer cell proliferation. (a) Promotion of gastric and colorectal cancer cell proliferation by H19. (b) Inhibition of gastric and pancreatic cell proliferation by GAS5; CRC: colorectal cancer, GC: gastric cancer, HCC: hepatocellular carcinoma, and PC: pancreatic cancer.

lncRNAs play important roles at initial stages of GI tract tumorigenesis by either promoting or repressing cancer cell proliferation (Figure 1). The lncRNA, GHET1, is involved in cell proliferation in GC. Yang et al. demonstrated that enhanced expression of GHET1 promotes cell proliferation both* in vitro* and* in vivo*, whereas the inhibition of GHET1 expression* via* siRNA, hinders cell proliferation* in vitro*. In a series of experiments, it was shown that GHET1 exerts its proproliferative role through upregulation of c-Myc expression, by binding IGF2BP1 and enhancing the interaction between IGF2BP1 protein and c-Myc mRNA [43]. H19 is another lncRNA, which affects cancer cell proliferation. The H19 gene is paternally imprinted and is expressed by the maternal allele. Loss of H19 imprinting [44] and overexpression was reported in several GI cancers such as GC, hepatocellular (HCC), and esophageal carcinomas [45]. Several mechanisms have been proposed for the oncogenic activity of H19 in GI cancers. In GC cell lines, ectopic expression of H19 results in increased cell proliferation and depletion of its expression promotes cell apoptosis. The mechanism underlying these observations was found to be the association of H19 with p53, which in turn affects the tumor suppressive function of the latter [46]. Additionally, a different study revealed that H19 expression can be induced by the oncogene c-Myc* in vitro*, and in GC tissue samples the expression of H19 positively correlates with c-Myc levels. The same research group revealed that depletion of H19 results in decreased proliferation of GC cells [47]. Furthermore, H19 is the primary precursor of miR-675, which in turn acts as the mediator of the H19 tumorigenic function in CRC cells* via* targeting the tumor suppressor protein RB [45]. Similarly to H19, c-Myc can also induce the transcription of CCAT1, also known as CARLo-5 (cancer-associated region long noncoding RNA), transcription and its ectopic expression was able to promote CRC cell proliferation* in vitro* [48]. CARLo-5 expression is regulated by a physical interaction between the* MYC* enhancer region and the active regulatory region of the CARLo-5 promoter. By performing siRNA-mediated silencing of CARLo-5 in various CRC cell lines, Kim et al. suggested that CARLo-5 inhibits G1 phase arrest by regulating the expression of several cell-cycle-related genes such as* CDKN1A* [49]. A similar study in GC cells, confirmed the regulatory role of CARLo-5 in cell proliferation and apoptosis. Briefly, it was found that knockdown of CARLo-5 resulted in decreased expression of PCNA (proliferating cell nuclear antigen), which is essential for DNA replication and increased synthesis of the cell-cycle regulators p16, p21, and p27. Moreover, siCARLo-5 cells were characterized by common features of late apoptosis and altered expression profiles of apoptosis-related proteins [50]. Additionally, the lncRNA HEIH plays an important role in cell-cycle regulation. In HCC cells, depletion of HEIH resulted in reduced cell proliferation and contributed to cell-cycle arrest mainly by p16, p27, and p21 protein upregulation. It was postulated that the association of HEIH with the enhancer of zeste homolog 2 (EZH2) is required for the repression of the EZH2 target genes, which include cell-cycle regulation genes [51]. HULC is a strongly upregulated mRNA-like lncRNA in liver cancer that localizes to the cytoplasm and is associated with ribosomes. Interestingly, knockdown of HULC RNA in HCC cells results in a significant up- or downregulation of different genes that have been previously reported to be involved in liver carcinogenesis [52]. HULC can also repress the expression of the tumor suppressor p18, enhancing in this way HCC proliferation mediated by Hepatitis B virus X protein (HBx) [53]. The transcription factor CREB can activate HULC expression in a cancer-specific manner through a regulatory loop which involves miR-372 and its target gene cAMP-dependent protein kinase catalytic subunit b (PRKACB) [54]. Additionally, knockdown of PVT-1 inhibits cell proliferation* in vitro* and promotes apoptosis through the activation of TGF-*β* signalling pathway-related genes such as SMAD4 [55]. Wang et al., have shown that RNA interference-mediated silencing of MALAT1 in GC cells, results in cell cycle arrest in G0/G1 phase and suppression of cell proliferation. MALAT1 was found to affect GC cell proliferation partly through the modulation of expression levels and nuclear distribution of SF2/ASF, a member of the serine/arginine (SR) splicing factors. This observation, in addition to the fact that MALAT1 is localized in nuclear speckles, implies that it may affect the alternative splicing of pre-mRNAs, through the modulation of SR factors [56].

On the contrary, overexpression of GAS5 can reduce GC cell proliferation and induce apoptosis both* in vitro* and* in vivo*, whereas knockdown of GAS5 can promote cell proliferation. Interestingly, inhibition of GAS5 expression resulted in increased protein levels of E2F1 and Cyclin D1, which are two major players in the retinoblastoma protein (pRB) pathway, as well as in decreased P21 levels which has a critical role in cell cycle arrest [57]. In pancreatic cancer cells, inhibition of GAS5 expression increases CDK6 protein levels thereby facilitating cell cycle progression [58]. MEG3 is abundantly expressed in normal human tissues and its expression is often lost in cancer, suggesting that it functions as a tumor suppressor lncRNA. Transfection of GC and CRC cells with MEG3 resulted in inhibition of cell proliferation and promotion of apoptosis, partly through activation of p53 [59, 60]. Ectopic expression of MEG3 in nude mice confirmed the tumor suppressive nature of this lncRNA, since MEG3 upregulation resulted in inhibition of CRC proliferation [60]. Further* in vitro* studies indicate that overexpression of MEG3 activates p53 and is able to inhibit cell proliferation, through the stimulation of endogenous p53 target genes [61]. In a series of* in vitro* experiments, Braconi et al. demonstrated that HCC cells with enforced MEG3 expression, have reduced capacity for anchorage-dependent and -independent growth; MEG3 expression induced also apoptosis in these cell lines. The same study showed that hypermethylation of MEG3 promoter is the main mechanism behind the decreased expression of MEG3 in HCC [62]. In GC, it was shown that miR-148a can indirectly induce the overexpression of MEG3, through downregulation of DNA methyltransferase 1, thereby inhibiting GC cell proliferation [63]. Moreover, loc285194 which is a p53-regulated tumor suppressor lncRNA was found to inhibit CRC cell growth both* in vitro* and* in vivo* [64].

**Figure 2:**
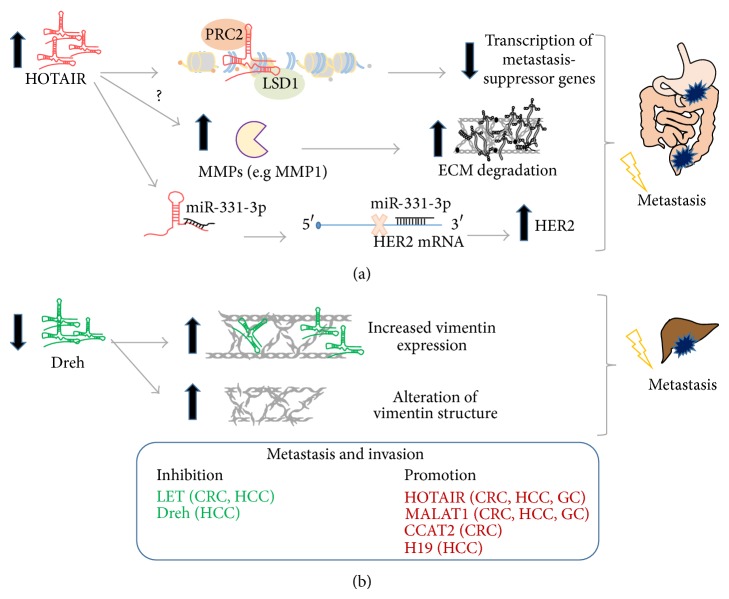
Representative mechanisms of lncRNAs implication in gastrointestinal cancer invasion and metastasis. (a) Promotion of gastric and colorectal cancer invasion and metastasis by HOTAIR. (b) Inhibition of hepatocellular carcinoma cell invasion by Dreh; CRC: colorectal cancer, GC: gastric cancer, HCC: hepatocellular carcinoma, and PC: pancreatic cancer.

lncRNAs are key players in the complex and multistep process of cancer progression, invasion, and metastasis (Figure 2). HOTAIR epigenetically regulates the expression of essential metastasis-suppressor genes through the coordination of histone modification complexes, PRC2 and LSD1. In CRC, HOTAIR closely correlates with members of the PRC2 complex and ectopic overexpression of HOTAIR promoted invasion of cancer cells* in vitro* [65]. Additionally, HOTAIR may promote migration and invasion of HCC cells, partially through the negative regulation of RNA binding motif protein 38 (RBM38) [66]. A different mechanism for HOTAIR oncogenic activity was provided by a recent study using* in vitro* assays in HCC. Silencing of HOTAIR expression reduced HCC cell proliferation and negatively regulated the expression levels of matrix metalloproteinase-9 (MMP9) and angiogenic factor VEGF, which are known to be involved in metastasis and angiogenesis, respectively [67]. Likewise, in GC cell lines, knock-down of HOTAIR, reduced invasiveness and the expression of MMP1 and MMP3. Another interesting finding was that HOTAIR silencing reversed EMT through regulation of Snail, which is one of the main transcription factors that controls EMT and cell motility [31]. HOTAIR can also act as an endogenous miRNA “sponge” which restrains miR-331-3p activity on HER2, thus enhancing HER2 oncogenic activity in GC cells [68]. Ma et al. showed that in gallbladder cancer cells HOTAIR is a direct target of c-Myc and its activity may be partially explained by the downregulation of miR- 130a,* via* promoter methylation [69]. MALAT1 is one of the most prominent oncogenic lncRNAs and is reported to be highly expressed in several GI cancers such as GC, CRC, and HCC. A number of studies provided evidence that ectopic expression of MALAT1 promotes proliferation and migration of cell lines* in vitro* and enhances tumor growth and metastasis* in vivo*. In CRC cells, MALAT1 was found to bind the tumor suppressor SFPQ protein, which in turn leads to release of the oncogenic PTBP2 from the SFPQ/PTBP2 complex, thereby promoting cell proliferation and migration. Additionally, the same research group showed that MALAT1 could promote metastasis of CRC cells in nude mice [70]. The abovementioned effects of MALT1 were confirmed by a recent study where the newly identified mechanism for gene induction, named RNAa, was used. According to these results, MALAT1 upregulation induces CRC cell growth and cell cycle G1/S phase transition* in vitro* and promotes CRC growth* in vivo*. Additionally, using orthotopic tumor models in nude mice, it was found that MALAT1 induction resulted in a significant increase of tumor metastatic potential. These effects were attributed to the induction of MALAT1 target gene* AKAP-9* [71]. The overexpression of CCAT2 promotes tumor growth and metastasis in CRC mouse xenograft models. It was demonstrated that CCAT2 directly interacts with the transcription factor TCF7L2, thereby activating WNT signaling pathway, while CCAT2 is also a downstream target of WNT. Additionally, CCAT2 can increase the expression of WNT target genes including MYC and MYC-regulated miRNAs such as miR-17-5p, and miR-20a, by TCF7L2-mediated transcriptional regulation [72]. Moreover, H19 is highly expressed in liver cancer cell lines, and hypoxic conditions strongly upregulate H19 levels. Tumor hypoxia affects cancer progression and metastasis, and it renders tumors resistant to anticancer therapy [73]. In contrast with the above, Zhang et al. demonstrated that H19 may activate miR-200 family members, resulting in subsequent suppression of their target genes, E-cadherin transcriptional repressors (ZEB1/2). The miR-200 family plays an important role in repression of EMT, through direct targeting of ZEB1/2 [74].

On the other side of the spectrum, LET acts as an inhibitor of metastasis in HCC under conditions of hypoxia. This lncRNA is downregulated in certain GI cancers, including HCC and CRC and its mechanism of action has been studied in orthotopic tumor models in nude mice. According to these results LET is a component of a positive feedback loop which includes the hypoxia-inducible factor 1, alpha subunit (HIF-1a) the histone deacetylase 3 (HDAC3), and the NF90. Upon hypoxia, LET is suppressed by HDAC3, and LET-NF90 interaction is disturbed. As a consequence, the stabilization of NF90 is enhanced, resulting in increased expression of HIF-1a, which has a well-documented role in hypoxia-induced invasion and metastasis [75]. The depletion of Dreh expression is found to be associated with increased invasion* in vitro* and* in vivo*. A mechanistic study showed that Dreh exerts its action through the binding of vimentin, a major cytoskeletal protein, thus leading to arrest of metastasis. In Dreh-transfected cells, repression of vimentin expression and changes regarding the vimentin organization and localization have been reported, which are related with inhibition of cell migration [76]. LEIGC (lower expression in gastric cancer) is a newly identified lncRNA with a tumor suppressive role in GC, which prevents cell migration and EMT* in vitro*. In more details, Han et al. performed gain- and loss-of-function experiments in GC cell lines and found that LEIGC silencing resulted in increased migration potential of the cells and was accompanied by morphological characteristics which are indicative of trans-differentiation from epithelial to mesenchymal phenotype. The latter was confirmed by both decreased expression of epithelial cell-related genes and proteins, such as* CDH1* and E-cadherin, and upregulation of mesenchymal-cell markers like* snail*,* slug*,* twist* and* zeb*, and vimentin [77].

Given the active involvement of lncRNAs in cancer progression, along with their aberrant expression which is often associated with clinicopathological characteristics and prognosis of cancer patients and their tissue- or cancer-specific expression patterns, it is probable that lncRNAs could soon outperform the currently suggested mRNA or protein markers. lncRNAs have just begun to prove their dynamics as diagnostic, prognostic, and predictive molecular markers for GI malignancies and further elucidation of specific mechanisms of action can pave the way for designing novel anticancer therapeutic approaches.

### 5.2. lncRNAs as Biomarkers for Colorectal Cancer

#### 5.2.1. lncRNAs in CRC Screening and Diagnosis

A number of lncRNAs may serve as molecular markers for CRC diagnosis (Table 1). Graham et al. showed that CRNDE splice variants are upregulated in early stage neoplastic colorectal tissues including adenomas and adenocarcinomas. Additionally, CRNDE splice variant h expression levels could efficiently discriminate adenomas from normal tissues and measurement of CRNDE-h RNA levels in plasma samples using qPCR resulted in a sensitivity of 87% and specificity of 93%, for detecting CRC [78]. The diagnostic performance of a newly detected lncRNA ncRuPAR was recently studied by Yan et al. The expression of ncRuPAR is downregulated in cancer and can effectively differentiate CRC from benign tissues, with high sensitivity [79].

CCAT1 has also been proposed as a potential biomarker for screening and diagnosis of CRC. This lncRNA is found to be overexpressed in tissues and in peripheral blood samples obtained from CRC patients compared to healthy controls [80]. Moreover, CCAT1 expression was studied across a spectrum of tissues from different stages of CRC progression. CCAT1 upregulation is evident in premalignant conditions and in all disease stages including distant CRC metastasis, suggesting that CCAT1 can also be used for monitoring of disease progression [81]. Interestingly, Kam et al. developed a CCAT1-specific peptide nucleic acid (PNA) based molecular beacon and showed that it can serve as a valuable tool for both imaging and* in situ* detection of CRC [82]. Single nucleotide polymorphisms (SNP) in lncRNA genes are associated with CRC susceptibility. In particular, two SNPs located in the lncRNA PRNCR1 gene (rs13252298 and rs1456315) are connected with decreased risk for CRC, while patients harboring two different SNPs, namely, rs7007694C and rs16901946G, have low risk to develop poorly differentiated CRC. On the contrary, the rs1456315G SNP is linked to increased risk for the development of CRC with poor differentiated status [83]. Similarly, Xue et al. reported that a HOTAIR SNP is associated with risk of CRC development. Briefly, the rs7958904 CC genotype is related with decreased risk of CRC compared to the rs7958904 GG genotype [84].

#### 5.2.2. lncRNAs in CRC Prognosis

A recent study demonstrated that increased HOTAIR expression, as assessed by qPCR in CRC tissues, positively correlates with advanced tumor stage, high recurrence rate, and short metastasis-free and OS intervals of the patients [65, 85]. Additionally, HOTAIR overexpression in blood samples obtained from CRC patients is significantly associated with reduced OS [86], a fact that strengthens HOTAIRs' vast potential as a prognostic biomarker. The prognostic significance of MALAT1 in stage II/III CRC patients is also revealed. Patients with elevated MALAT1 expression have significantly higher risk for metastasis after radical surgery, and MALAT1 expression is an independent prognostic factor of both disease-free survival (DFS) and OS of CRC patients [87]. Furthermore, PVT1 expression is found to be a powerful prognostic indicator in CRC. Patients with high PVT1 expression levels present a more adverse outcome as indicated by shorter OS periods, compared to those with low PVT1 expression. Moreover, multivariate analysis revealed that PVT1 expression in CRC predicts an increased risk of death, independently of important clinicopathological factors [55]. UCA1, exhibits high expression in CRC tissues and correlates with larger tumor size and unfavorable prognosis of the CRC patients [88]. Similarly, overexpression of PCAT-1 is an indicator of distant metastasis and an independent predictor of poor OS of patients [89]. The levels of lncRNA 91H expression are significantly upregulated in CRC compared to noncancerous tissues, and 91H constitutes an independent predictor of poor prognosis in CRC [90]. The expression of BANCR is positively associated with lymph node metastasis and tumor stage; however, its prognostic significance, in terms of patients' survival, has not yet been reported [91]. LSINCT5 upregulation is associated with more aggressive disease phenotypes as well as with shorter DFS and DSS periods, revealing its possible role as an indicator of poor prognosis in CRC [92].

On the other hand, the expression levels of numerous lncRNAs are reported to be downregulated in CRC. Reduced expression levels of LOC285194 are associated with more aggressive features of tumors and correlate significantly with shorter DFS [93]. Low ncRAN levels are detected in high histological grade tumors and more importantly decreased expression of this lncRNA seems to be an independent prognostic factor of poor OS [94]. Focusing on RP11-462C24.1, low expression levels are found in patients with metastasis and multivariate analysis revealed that decreased RP11-462C24.1 expression can serve as an independent prognosticator of poor DSS of CRC patients [95]. The expression of a novel class of lncRNAs, transcribed ultraconserved regions (T-UCRs), namely, uc.73 and uc.388, is reported to decrease in CRC and uc.73 is found to be associated with OS of CRC patients [96]. Additionally, patients with low GAS5 expression had significantly shorter OS than those with high GAS5 expression and GAS5 expression was identified as an independent indicator of CRC prognosis [97]. Likewise, MEG3 downregulation is associated with advanced TNM stages, deeper tumor invasion and inferior OS intervals. Moreover, MEG3 is an independent indicator of favorable prognosis in terms of OS in CRC patients [60].

Recently, a prognostic lncRNA signature consisting of six different lncRNAs was identified by Hu et al., using a microarray data mining approach and CRC data sets from GEO database. Based on this signature they divided CRC patients into high- and low-risk groups and showed that patients belonging to the former group had shorter DFS [98].

### 5.3. lncRNAs as Biomarkers for Gastric Cancer

#### 5.3.1. lncRNAs in GC Diagnosis

Many studies imply the presence of differential levels of several lncRNAs between cancerous and noncancerous conditions, not only in tissues but in biofluids as well [46, 57, 59, 68, 99–113] (Table 1). Arita et al. showed not only a significant overexpression of H19 in the plasma of GC patients compared to healthy controls, but also a substantial reduction of the levels of circulating H19 in plasma after gastrectomy [114]. The expression levels of Linc00152 were analyzed in gastric juice from GC patients and normal controls and a significant increase of Linc00152 in patients was observed [115]. AA174084 is another lncRNA with possible diagnostic value in GC, as it was significantly upregulated in gastric juice from GC patients compared to the gastric juice derived from patients with other gastric disorders or to normal mucosa [110]. According to the analysis of global expression profile of lncRNAs in GC, many lncRNAs exhibited different expression patterns in paired GC and normal adjacent tissue samples [116, 117]. Recently, Mizrahi et al. compared the expression levels of CCAT1 in GC tissues to normal ones, obtained from patients with morbid obesity, revealing a statistical significant increase in GC patients [118].

#### 5.3.2. lncRNAs in GC Prognosis

HOTAIR, seems to be an indicator of poor prognosis in GC patients, since its overexpression was associated with advanced TNM and pathological stage, large tumor size, increased tumor invasiveness, and metastatic potential as well as with an adverse outcome in terms of OS [68, 99–103]. Moreover, increased expression of H19 was significantly associated with shorter OS periods; highlighting its value as a biomarker of unfavorable prognosis [104]. A recent study revealed that ANRIL upregulation is an independent marker of unfavorable prognosis in GC, since its expression positively correlates with advanced disease stage and large tumor size as well as with limited DFS and OS [105]. CCAT1 and CCAT2 are also considered as indicators of poor prognosis. Specifically, high expression levels of CCAT1 were associated with tumor growth, lymph node metastasis [119], and advanced TNM stage [50], whereas CCAT2 overexpression was related with increased metastatic and invasive potential of the tumor. Moreover CCAT2 is an independent prognostic indicator of decreased DFS and OS intervals in GC patients [120]. Similarly, HULC upregulation was associated with increased tumor metastatic potential, unraveling the role of this lncRNA as an indicator of poor prognosis [106]. LSINCT5 is also an independent marker of unfavorable prognosis and its expression positively correlates with several clinicopathological parameters of poor prognosis in GC (advanced TNM stage, large tumor size, lymph node metastasis, and deeper invasion depth) as well as with shorter DFS and DSS periods [92]. Finally, overexpression of GHET1 is significantly related to tumor size and invasion and to poor OS of the patients [43].

On the other hand, lncRNAs such as FENDRR, MEG3, LET, GAS5, BM742401, and GACAT1 were found to represent indicators of favorable prognosis. In particular, FENDRR decreased expression seemed to be an independent prognostic indicator of decreased DFS and OS intervals and was associated with deeper tumor invasion, advanced tumor stage, and lymph node metastasis [111]. Concerning MEG3, its downregulation was associated not only with markers of poor prognosis, namely, large tumor size, advanced pathological stage and increased depth of invasion, but also with shorter OS [59]. Similarly, LET expression was negatively associated with tumor invasiveness and TNM stage, and decreased LET expression levels consist an independent prognostic marker of limited OS of GC patients [121]. GAS5 and BM742401 are additional markers of favorable GC patients' prognosis since decreased expression levels were associated with poorer survival [57, 122]. In advanced disease stages, the downregulation of GACAT1 was related with increased tumor invasiveness and metastatic potential [112] (Table 1).

### 5.4. lncRNAs as Biomarkers for Hepatocellular Carcinoma (HCC)

#### 5.4.1. lncRNAs in HCC Diagnosis

Xie et al. reported that HULC plasma levels were significantly higher in HCC patients than in the healthy control group and that HULC tissue expression demonstrated adequate sensitivity and specificity to discriminate malignant from normal liver tissues [123]. Additionally, HULC seems to be a hepatocarcinogenesis-specific lncRNA since it is only slightly overexpressed in cirrhotic liver disorders. Recently, it was reported that lncRNA-uc003wbd and lncRNA-AF085935 serum levels can effectively discriminate HCC from HBV infection as well as HCC and HBV-affected patients from healthy volunteers [124]. lncRNAs such as HULC, HOTAIR, H19, HEIH, MVIH, and PVT1 are upregulated in HCC, in contrast to MEG3, hDREH, GAS5, and LET that exhibit lower expression in this malignancy [125]. Particularly, HEIE seems to be linked with pathological conditions of liver, since it was found to be overexpressed both in cancer and cirrhosis [51]. According to a recent microarray-based analysis of the expression profile of lncRNAs in HCC, 214 lncRNAs exhibited aberrant expression in HCC tissues compared to normal ones [126].

#### 5.4.2. lncRNAs in HCC Prognosis

HULC is a marker of poor prognosis in HCC since patients with higher Edmondson grades or with positive HBV (Hepatitis B Virus) status demonstrated higher HULC levels both in plasma and tissue [123]. HOTAIR upregulation was associated with lymph node metastasis [67] and large tumor size [127] as well as inferior DFS after resection or transplantation [125], revealing its potential as marker of unfavorable patients' prognosis. HEIH is an independent prognostic indicator of shorter OS and DFS in HBV-related HCC [51], whereas MVIH is an independent marker of poor outcome after hepatectomy, as its upregulation is related to lower DFS and OS probabilities [128]. According to recent data, four additional lncRNAs, namely, URCH, lncRNA-ATB, HOTTIP, and PVT1, seem to have a role as markers of poor prognosis. Specifically, increased URCH levels were associated with inferior OS intervals [129]; upregulation of lncRNA-ATB exhibited a strong association with liver cirrhosis and microvascular and macrovascular invasion and encapsulation [130] and HOTTIP levels, along with the mRNA HOXA13, were associated with patients' clinical progression and were able to predict disease outcome [131]. Regarding PVT1, its expression levels were analyzed in tissues obtained from patients subjected to liver transplantation, and significant associations of PVT1 overexpression with advanced TNM stages, higher recurrence rate, and increased AFP levels were observed. Additionally, PVT1 seems to be an independent predictor of poor DFS in HCC patients after liver transplant [132].

As opposed to the lncRNAs discussed above, hDREH, LET, and GAS5 may hold value as biomarkers of favorable prognosis for HCC. In particular, hDREH downregulation is associated with shorter DFS and OS intervals of HCC patients [76]. Moreover, LET decreased expression was associated with tumor micrometastasis and encapsulation [125], whereas GAS5 downregulation was associated with markers of poor prognosis (tumor size, lymph node metastasis, and clinical stage) and emerged as an independent predictor of 5-year DFS [133] (Table 2).

### 5.5. lncRNAs as Prognostic Biomarkers for Pancreatic Cancer

The clinical relevance of lncRNA MALAT1, in pancreatic ductal adenocarcinoma (PDAC), was investigated by Liu et al. The expression of MALAT1 was measured in FFPE tissues using qPCR and was found to be significantly higher in PDAC compared to adjacent noncancerous tissues. Additionally, MALAT1 expression levels were positively correlated with tumor size, stage, and depth of invasion and the overexpression of MALAT1 was independently associated with poorer disease-specific survival of PDAC patients [134]. In concordance with the aforementioned results, Pang et al. reported that MALAT1 overexpression in pancreatic cancer tissues is related to aggressive phenotypes of the disease and is an independent marker of poor prognosis regarding OS [135]. HOTAIR is also highly expressed in aggressive pancreatic tumors and the respective patients had significantly shorter OS [136]. Furthermore, lncRNA PVT1 is an independent marker of decreased OS in PDAC and it is associated with clinical stage and N-classification [137].

A novel lncRNA ENST00000480739 was recently identified through lncRNA expression microarray. The expression of this lncRNA is significantly lower in PDAC patients with lymph node metastasis and its expression is associated with prolonged OS [3]. Likewise, the lncRNA BC008363 is found to be downregulated in PDAC compared to paired noncancerous tissues. Patients with high expression levels had significantly better OS, suggesting that BC008363 is a biomarker of favorable prognosis in PDAC [138]. Several lncRNAs are implicated in metastasis in pancreatic cancer, suggesting that they are associated with unfavorable prognosis of patients. For instance, Tahira et al. identified a set of 134 lncRNAs that correlate with metastasis in PDAC [139]. A different study revealed that H19 is overexpressed in PDAC tissues and in primary pancreatic tumors that subsequently metastasize [140] (Table 2).

### 5.6. lncRNAs as Biomarkers for Esophageal Cancer

#### 5.6.1. lncRNAs in Esophageal Cancer Screening and Diagnosis

Esophageal cancer (EC) occurs in two main histological subtypes, esophageal squamous cell carcinoma (ESCC) and esophageal adenocarcinoma (EAC), which differ in incidence and etiology. Several lncRNAs are reported to be aberrantly expressed in esophageal cancer. For instance, the expression of ANRIL [141] and POU3F3 [142] is found to be higher in ESCC compared to noncancerous tissues. Interestingly, Tong et al. reported a significant increase of POU3F3 plasma levels in ESCC patients compared to healthy controls. They also found that the combination of POU3F3 with the classic tumor marker, SCCA, can aid in early detection of ESCC, revealing its role as a promising diagnostic tool for population screening [143]. Furthermore, a functional genetic polymorphism in lincRNA-uc003opf.1 exon was found to be associated with susceptibility to ESCC. In particular, patients with rs11752942AG and GG genotypes have lower risk for ESCC compared to rs11752942AA, revealing their value as markers for screening high-risk populations [144]. In EAC, HNF1A-AS1 is strongly upregulated in cancerous tissues compared to corresponding normal ones, revealing its possible diagnostic value for this subtype of esophageal cancer [145]. The expression of AFAP1-AS1 is markedly increased in EAC compared to normal esophageal tissues and hypomethylation is responsible for the observed upregulation [146]. Recently, Fassan et al., using microarray analysis, identified T-UCR signatures that were deregulated across the squamous epithelia to Barrett's adenocarcinoma sequence of neoplastic progression, suggesting that these signatures may serve as tools for assessing risk of developing Barrett's-esophagus associated cancer [147].

#### 5.6.2. lncRNAs in Esophageal Cancer Prognosis and Treatment Response Prediction

Similarly to other GI cancers, an important correlation with limited DFS and OS of ESCC patients has been observed for HOTAIR expression [148]. A different study showed that increased HOTAIR expression is also associated with advanced TNM stage ESCC tumors and lymph node metastasis, a fact that strengthens its potential as a biomarker of poor prognosis [149]. Moreover, the association of a newly identified lncRNA FOXCUT with shorter survival time of patients after surgery, poorly differentiated tumors and metastasis, indicates its significant value as a biomarker of unfavorable prognosis of ESCC patients [150]. According to recent data, UCA1, a lncRNA with oncogenic role in ESCC, is an independent predictor of shorter OS and is related to advanced tumor stage and differentiation grade, increased lymphatic invasion as well as poor survival rates [151]. Two additional lncRNAs, SPRY4-IT1 and PCAT-1, may serve as indicators of unfavorable prognosis in ESCC since their expression levels were associated with markers of poor prognosis, such as lymphatic invasion, and shorter OS [152, 153]. SPRY4-IT1 is also an independent prognostic marker of ESCC patients' OS [152]. Both PEG10 and TUG1 lncRNAs exhibit tumorigenic potential in EC, and recently they were found to be overexpressed in cancerous tissues compared to normal ones. It was also reported that expression levels of lncRNA PEG10 were positively correlated with lymph node metastasis and tumor differentiation status [154], whereas higher TUG1 expression levels were observed in patients with family history of EC as well as with upper segment of ESCC [155]. High PlncRNA-1 expression is found in advanced stage tumors (stage III/IV) and in tumors that spread to lymph nodes, suggesting that it may serve as a molecular marker of poor prognosis [156].

On the contrary, the expression of 91H is negatively associated with depth of tumor invasion, tumor grade, and TNM stage, demonstrating its favorable prognostic impact in ESCC [157]. Moreover, Tong et al. performed expression analysis of LOC285194 using ESCC tissue specimens from of patients prior to treatment, after chemoradiotherapy (CRT) and surgical resection, and from patients who underwent esophagectomy alone, in order to unravel the prognostic and predictive significance of this lncRNA. Interestingly, low LOC285194 expression was associated with adverse clinicopathological characteristics of the tumors such as TNM stage and distant metastasis. In the group of patients who received CRT, the expression of LOC285194 was found to be an independent predictor of patients' response to therapy, and in the subgroup patients after esophagectomy the LOC285194 levels were found to significantly correlate with their improved DFS and OS probabilities [158] (Table 3).

## 6. lncRNAs as Therapeutic Modulators or Targets in Digestive System Malignancies

The most prominent example of the utility of lncRNAs as therapeutic tools in targeted anticancer therapy is the construction of a DNA vector, namely, BC-819 (DTA-H19) which carries the diphtheria toxin A (DTA) gene under the control of transcriptional regulatory sequences of H19. The BC-819 plasmid takes advantage of the tumor specific expression of H19 and acts as a “trojan horse” to kill cancer cells through the expression of DTA [44]. The safety and efficiency of BC-819 vector is evaluated in clinical trials and so far phase I/II studies have been completed in pancreatic cancer [40, 44].

The use of small interfering RNAs (siRNA) to inhibit lncRNAs is another approach with promising results regarding targeted therapy of GI cancers, although only* in vitro* data are available so far. In liver cancer cell lines, HOTAIR silencing inhibited cell proliferation and increased the cells sensitivity to Cisplatin and Doxorubicin [125]. MRUL (multidrug resistant-related and upregulated lncRNA) is overexpressed in two MDR GC cell sublines, and depletion of MRUL expression* via* siRNA, increased chemosensitivity of GC cells to Adriamycin and Vincristine [159]. Recently, a significant downregulation of snaR was observed in two 5- fluorouracil (FU) resistant colon cancer cell lines (SNU-C4R and SNU-C5R) and siRNA silencing of this lncRNA resulted in increased cell viability after treatment with 5-FU [160].

Modulation of the expression of certain tumor-suppressor lncRNAs can be used as an alternative therapeutic approach in GI cancers. For instance, H19 upregulation suppressed* MDR1* expression and sensitized the cells to Doxorubicin* in vitro* [161]. Furthermore, PVT1 overexpression in pancreatic cancer cells resulted in resistance to Gemcitabine [162]. Targeting of certain miRNA-lncRNA interaction axes holds promise as a therapeutic strategy in HCC and GC patients [163]. In particular, targeting of HOTAIR-miR-331-3p-HER2 axis is a promising therapeutic perspective for HER-2 positive GC patients [68].

## 7. Conclusions

lncRNAs are steadily becoming one of the current trends in cancer research. In less than two years (2013–2015), an estimated total of >700 articles regarding the mechanistic and/or clinical role of lncRNAs in human malignancies has been published. It is expected that the profound implication of lncRNAs in cancer dynamics will be effectively translated into clinical practice through a novel class of biomarkers for major gastrointestinal malignancies. In fact, lncRNAs are endowed with certain characteristics that make them ideal as novel cancer biomarkers: (i) due to their broad implication in cancer pathobiology, a single deregulated lncRNA molecule can reflect changes in multiple cancer-affected pathways, (ii) they can be assayed in a wide variety of biological specimens, apart from tissues, such as serum or gastric fluid, where they show enhanced stability, (iii) they can be determined at low cost and can be easily assessed by simple methodologies such as qPCR that have already been introduced in routine clinical practice. Regarding the clinical management of GI cancers (Tables 1–3) some of the most promising lncRNA-oriented studies include: (i) the development of a CCAT1-specific peptide nucleic acid based molecular beacon for both imaging and* in situ* detection of CRC [82], (ii) the diagnostic value of HULC serum levels in HCC [123], (iii) the prognostic potential of HOTAIR for all GI cancers [86, 99, 103, 114, 127, 136, 149], and (iv) the predictive role of LOC285194 regarding chemoradiotherapy response in esophageal cancer [158]. However, there is an imperative need for validation of the biomarker potential of lncRNAs in GI malignancies, since up to now only the prognostic role of HOTAIR in GC [101, 103, 114] and CRC [65, 85, 86] has been demonstrated by at least two independent studies; in this respect, validation in prospective cohort studies could provide higher levels of evidence and accelerate the final clinical evaluation of specific lncRNAs as biomarkers.

Although the implication of lncRNAs in GI cancer is a fast expanding field of research, there are still many gaps that need to be filled. A further unraveling of the molecular functions of lncRNAs will significantly aid in the clarification of the genetic and molecular basis of cancer and is bound to provide novel lncRNA based therapeutics, apart from the ones currently being investigated in clinical trials for pancreatic cancer [44, 164]. The elucidation of miRNA-mRNA-lncRNA axes of interaction, the deciphering of lncRNA gene methylation patterns, the characterization of SNPs affecting lncRNA expression levels, and/or function in GI malignancies could form the starting point in the identification of a novel therapeutic class of molecules for major digestive system malignancies.

## Figures and Tables

**Table 1:** An overview of the biomarker potential of lncRNAs for colorectal and gastric cancer.

lncRNA	Cancer type	Sample	Expression	Clinical application	Reference
CRNDE (splice variants)	CRC	Tissue	Up	Diagnosis	[78]

CRNDE h	CRC	Plasma	Up	Diagnosis	[78]

ncRuPAR	CRC	Tissue	Down	Diagnosis	[79]

MALAT1	CRC	Tissue	Up	Unfavorable prognosis	[87]

PVT1	CRC	Tissue	Up	Unfavorable prognosis	[55]

UCA1	CRC	Tissue	Up	Unfavorable prognosis	[88]

PCAT-1	CRC	Tissue	Up	Unfavorable prognosis	[89]

91H	CRC	Tissue	Up	Unfavorable prognosis	[90]

LOC285194	CRC	Tissue	Down	Favorable prognosis	[93]

ncRAN	CRC	Tissue	Down	Favorable prognosis	[94]

RP11-462C24.1	CRC	Tissue	Down	Favorable prognosis	[95]

u.73	CRC	Tissue	Down	Favorable prognosis	[96]

H19	GC	Plasma	Up	Diagnosis	[114]
	GC	Tissue	Up	Unfavorable prognosis	[104]

Linc00152	GC	Gastric juice	Up	Diagnosis	[115]

AA174084	GC	Gastric juice	Up	Diagnosis	[110]

AC138128.1	GC	Tissue	Down	Diagnosis	[109]

ANRIL	GC	Tissue	Up	Unfavorable prognosis	[105]

GHET1	GC	Tissue	Up	Unfavorable prognosis	[43]

HULC	GC	Tissue	Up	Unfavorable prognosis	[106]

CCAT2	GC	Tissue	Up	Unfavorable prognosis	[120]

FENDRR	GC	Tissue	Down	Favorable prognosis	[111]

GACAT1	GC	Tissue	Down	Diagnosis/favorable prognosis	[112]

LET	GC	Tissue	Down	Favorable prognosis	[121]

BM742401	GC	Tissue	Down	Favorable prognosis	[122]

CCAT1 (CARLo-5)	CRC	Tissue/blood	Up	Screening/diagnosis/monitoring of disease progression	[81]
	GC	Tissue	Up	Unfavorable prognosis	[119]

HOTAIR	CRC	Tissue/blood	Up (blood)	Unfavorable prognosis	[65, 85, 86]
	GC	Tissue	Up	Unfavorable prognosis	[100, 101, 103]

LSINCT5	CRC	Tissue	Up	Unfavorable prognosis	[92]
	GC	Tissue	Up	Unfavorable prognosis	[92]

GAS5	CRC	Tissue	Down	Favorable prognosis	[97]
	GC	Tissue	Down	Favorable prognosis	[57]

MEG3	CRC	Tissue	Down	Favorable prognosis	[60]
	GC	Tissue	Down	Favorable prognosis	[59]

**Table 2:** An overview of the biomarker potential of lncRNAs for hepatocellular and pancreatic cancer.

lncRNA	Cancer type	Sample	Expression	Clinical application	Reference
HULC	HCC	Tissue/plasma	Up	Diagnosis/unfavorable prognosis	[123]

uc003wbd	HCC	Serum	Up	Diagnosis	[124]

AF085935	HCC	Serum	Up	Diagnosis	[124]

HEIE	HCC	Tissue	Up	Unfavorable prognosis	[51]

MVIH	HCC	Tissue	Up	Unfavorable prognosis	[128]

URCH	HCC	Tissue	Up	Unfavorable prognosis	[129]

lncRNA-ATB	HCC	Tissue	Up	Unfavorable prognosis	[130]

HOTTIP	HCC	Tissue	Up	Unfavorable prognosis	[131]

hDREH	HCC	Tissue	Down	Favorable prognosis	[76]

LET	HCC	Tissue	Down	Favorable prognosis	[125]

GAS5	HCC	Tissue	Down	Favorable prognosis	[133]

MALAT1	PDAC	FFPE tissues	Up	Unfavorable prognosis	[134, 135]

ENST00000480739	PDAC	Tissue	Down	Favorable prognosis	[3]

BC008363	PDAC	Tissue	Down	Favorable prognosis	[138]

HOTAIR	HCC	Tissue	Up	Unfavorable prognosis	[127]
	Pancreatic cancer	Tissue	Up	Unfavorable prognosis	[136]

PVT1	HCC	Tissue	Up	Unfavorable prognosis	[132]
	PDAC	Tissue	Up	Unfavorable prognosis	[137]

**Table 3:** An overview of the biomarker potential of lncRNAs for esophageal cancer.

lncRNA	Cancer type	Sample	Expression	Clinical application	Reference
HOTAIR	ESCC	Tissue	Up	Unfavorable prognosis	[148]

LOC285194	ESCC	Tissue	Down	Favorable prognosis and prediction of response to chemotherapy	[158]

POU3F3	ESCC	Plasma	Up	Diagnosis	[143]

UCA1	ESCC	Tissue	Up	Unfavorable prognosis	[151]

SPRY4-IT1	ESCC	Tissue	Up	Unfavorable prognosis	[152]

PCAT-1	ESCC	Tissue	Up	Unfavorable prognosis	[153]

## Abbreviations

91H: Antisense H19
AFAP1: Actin filament associated protein 1
AFAP1-AS1: AFAP1 antisense RNA 1
AFP: Alpha-fetoprotein
*AKAP-9*: A kinase (PRKA) anchor protein 9
ANRIL: Antisense noncoding RNA in the INK4 locus
ASF1: Alternative splicing factor 1
BANCR: BRAF-activated non-protein coding RNA
CA19-9: Carbohydrate antigen 19-9
CARLo-5: Cancer-associated region long noncoding RNA
CCAT1: Colon cancer associated transcript 1
CCAT2: Colon cancer associated transcript 2
*CDKN1A*: Cyclin-dependent kinase inhibitor 1A
CRC: Colorectal cancer
CREB: cAMP response element-binding protein
CRNDE: Colorectal neoplasia differentially expressed
CRNDE-h: CRNDE transcript variant h
DFS: Disease-free survival
Dreh: Downregulated expression by HBx
DSS: Disease-specific survival
DTA: Diphtheria toxin A
E2F1: E2F transcription factor 1
EAC: Esophageal adenocarcinoma
EC: Esophageal cancer
EMT: Epithelial-mesenchymal-transition
ESC: Embryonic stem cells
ESCC: Esophageal squamous cell carcinoma
EZH2: Enhancer of zeste 2 polycomb repressive complex 2 subunit
FDA: Food and Drug Administration
FENDRR: FOXF1 adjacent noncoding developmental regulatory RNA
FFPE: Formalin-fixed, paraffin-embedded (tissue)
FOXC1: Forkhead box C1
FOXCUT: FOXC1 upstream transcript
FU: Fluorouracil
GACAT1: Gastric cancer associated transcript 1 (previously known as AC096655.1-002)
GAS5: Growth arrest-specific 5
GC: Gastric cancer
GHET1: Gastric carcinoma proliferation enhancing transcript 1
GI: Gastrointestinal
GR: Glucocorticoid receptor
GRE: Glucocorticoid response elements
H19: Imprinted maternally expressed transcript
HBV: Hepatitis B virus
HBx: Hepatitis B virus X protein
HCC: Hepatocellular carcinoma
HDAC3: Histone deacetylase 3
HEIH: Hepatocellular carcinoma upregulated EZH2-associated long noncoding RNA
HIF-1a: Hypoxia-inducible factor 1, alpha subunit
HNF1A: Hepatocyte nuclear factor 1 homeobox A
HNF1A-AS1: HNF1A antisense RNA 1
HOTAIR: HOX transcript antisense intergenic RNA
HOTTIP: HOXA distal transcript antisense RNA
HOX: Homeobox
HULC: Highly upregulated in liver cancer
IGF2BP1: Insulin-like growth factor 2 mRNA binding protein 1
Kcnq1ot1: * Kcnq1* overlapping transcript 1
LC: Liver cancer
LEIGC: Lower expression in gastric cancer
LET: Low expression in tumor
lincRNAs: Long intergenic RNA
lncRNAs: Long noncoding RNAs
LSD1: Lysine-specific histone demethylase 1
MALAT1: Metastasis associated lung adenocarcinoma transcript 1
MAPK: Mitogen-activated protein kinase
MDR: Multiple drug resistance
MEG3: Maternally expressed 3
miRNA: MicroRNAs
MMP: Matrix metalloproteinase
MRUL: MDR-related and upregulated lncRNA
MVIH: Microvascular invasion in HCC
ncRAN: Noncoding RNA expressed in aggressive neuroblastoma
ncRuPAR: Nonprotein coding RNA, upstream of F2R/PAR1
NSCLC: Non-small cell lung carcinoma
ORFs: Open reading frames
OS: Overall survival
PANDA: P21 associated ncRNA DNA damage activated
PCA3: Prostate cancer associated 3
PCAT-1: Prostate cancer associated transcript 1
PCGEM1: Prostate-specific transcript 1
PCNA: Proliferating cell nuclear antigen
PDAC: Pancreatic ductal adenocarcinoma
PlncRNA-1: Prostate cancer-upregulated long noncoding RNA 1
POU3F3: POU class 3 homeobox 3
PRC2: Polycomb repression complex 2
PRKACB: cAMP-dependent protein kinase catalytic subunit b
PRNCR1: Prostate cancer associated noncoding RNA 1
PTBP2: Polypyrimidine tract binding protein 2
RB: Retinoblastoma
RBM38: RNA binding motif protein 38
RNAa: RNA activation
SCCA: Serum squamous cell carcinoma antigen
SF2: Pre-mRNA-splicing factor SF2
SFPQ: Splicing factor proline/glutamine-rich
siRNA: Small interfering RNA
SNPs: Single nucleotide polymorphism
SRA: Steroid receptor RNA activator
TCF7L2: Transcription factor 7-like 2
TERRA: Telomeric repeat-containing RNA
TGF-*β*: Transforming growth factor beta
TNM: Tumor-nodes-metastasis
T-UCR: Transcribed ultraconserved genomic region
TUG1: Taurine upregulated 1
UCA1: Urothelial cancer associated 1
URCH: Upregulated in hepatocellular carcinoma
VEGF: Vascular endothelial growth factor
XIST: X-inactive-specific transcript
Xlsirts: * Xenopus laevis* short interspersed repeat transcripts
ZEB1/2: Zinc finger E-box binding homeobox 1/2.
